# 

**Published:** 1906-04-28

**Authors:** 


					the hospital.
No. 1,022. Vol. XL. [afSS Saturday,
April 28th, 1906-
[SabscriptioiigTerms] pRICE J(|p??p?||Q?

				

